# Focus on the Role of NLRP3 Inflammasome in Diseases

**DOI:** 10.3390/ijms21124223

**Published:** 2020-06-13

**Authors:** Roberta Fusco, Rosalba Siracusa, Tiziana Genovese, Salvatore Cuzzocrea, Rosanna Di Paola

**Affiliations:** 1 Department of Chemical, Biological, Pharmaceutical and Environmental Sciences, University of Messina, Viale Ferdinando Stagno D’Alcontres, n 31, 98166 Messina, Italy; rfusco@unime.it (R.F.); rsiracusa@unime.it (R.S.); tgenovese@unime.it (T.G.); 2 Department of Pharmacological and Physiological Science, Saint Louis University School of Medicine, 1402 South Grand Blvd, St. Louis, MO 63104, USA

**Keywords:** inflammasome, metabolic pathologies, cardiovascular diseases, inflammatory issues, neurologic disorders

## Abstract

Inflammation is a protective reaction activated in response to detrimental stimuli, such as dead cells, irritants or pathogens, by the evolutionarily conserved immune system and is regulated by the host. The inflammasomes are recognized as innate immune system sensors and receptors that manage the activation of caspase-1 and stimulate inflammation response. They have been associated with several inflammatory disorders. The NLRP3 inflammasome is the most well characterized. It is so called because NLRP3 belongs to the family of nucleotide-binding and oligomerization domain-like receptors (NLRs). Recent evidence has greatly improved our understanding of the mechanisms by which the NLRP3 inflammasome is activated. Additionally, increasing data in animal models, supported by human studies, strongly implicate the involvement of the inflammasome in the initiation or progression of disorders with a high impact on public health, such as metabolic pathologies (obesity, type 2 diabetes, atherosclerosis), cardiovascular diseases (ischemic and non-ischemic heart disease), inflammatory issues (liver diseases, inflammatory bowel diseases, gut microbiome, rheumatoid arthritis) and neurologic disorders (Parkinson’s disease, Alzheimer’s disease, multiple sclerosis, amyotrophic lateral sclerosis and other neurological disorders), compared to other molecular platforms. This review will provide a focus on the available knowledge about the NLRP3 inflammasome role in these pathologies and describe the balance between the activation of the harmful and beneficial inflammasome so that new therapies can be created for patients with these diseases.

## 1. Mechanisms of NLRP3 Inflammasome Activation

**Figure 1:**
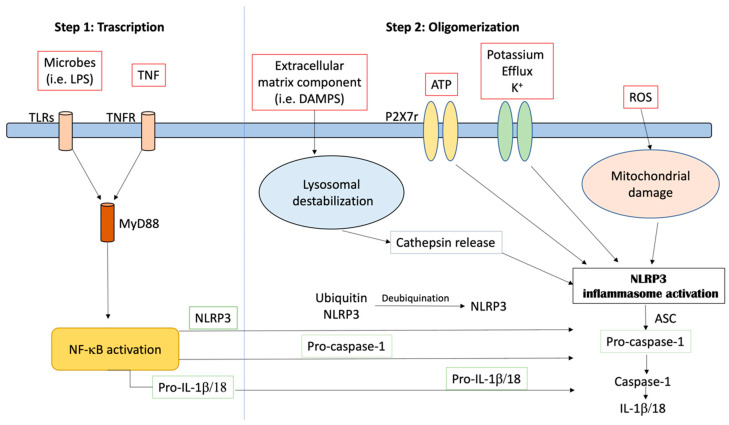
Activation of the NLRP3 inflammasome.

The NLRP3 inflammasome is a tripartite molecule of the nucleotide-binding domain and leucine-rich repeat (NLR) family, containing a nucleotide-binding NACHT domain with ATPase activity, an amino-terminal pyrin (PYRIN) domain and a carboxy-terminal leucine-rich repeat (LRR) domain [1]. NLRP3 controls the inflammatory response and coordinates the antimicrobial host. It responds to a diverse set of stimuli. These include extracellular ATP, particulate matter (such as silica, uric acid crystals, alum and asbestos) and crystalline, RNA–DNA hybrids, pore-forming toxins and numerous bacterial, fungal, protozoan and viral pathogens [2]. The activation of NLRP3 also needs priming by extracellular stimuli, which leads to the transcriptional induction of NLRP3 and post-translational modifications that permit receptor activation. Given the wide variety of stimuli, it is likely that NLRP3 responds to a common cellular event that is triggered by these activators, as shown in Figure 1. However, many different mechanisms have been proposed, including the production of reactive oxygen species, the release of oxidized mitochondrial DNA, lysosomal destabilization, mitochondrial dysfunction, the formation of large nonspecific membrane pores, changes in intracellular calcium levels, and potassium efflux [3]. In particular, it has been described that low potassium medium alone is sufficient to trigger NLRP3 activation [4]. Nowadays, two different types of NLRP3 activation have been described—canonical and non-canonical. Canonical NLRP3 inflammasome activation demands two independent and parallel steps—transcription (priming) and oligomerization (activation) [3]. In the first phase, innate immune signaling via cytokine receptors, such as the tumor necrosis factor (TNF) receptor and/or the toll-like receptor (TLR)-adaptor myeloid differentiation primary response 88 (MyD88), promotes NLRP3 and pro-IL-1β transcription through the nuclear factor-kB (NF-kB) activation. In the second phase, the oligomerization of the NLRP3 with an apoptosis-associated speck-like protein containing a CARD (ASC) leads to pro-caspase-1 activation and IL-18 and IL-1β release [3,5]. Different stimuli, including the inhibition of mitochondrial or glycolytic metabolism, viral RNA, extracellular osmolarity, β-amyloid (Aβ) protein and α-synuclein (α -syn) accumulation, post-translational NLRP3 alteration (such as ubiquitination and phosphorylation) and the degradation of extracellular matrix constituents may produce NLRP3 inflammasome activation and oligomerization. Moreover, the increase in potassium efflux through the cell membranes (i.e., exposure to pore-forming gasdermin D, mixed lineage kinase domain-like protein activation, P2X7 purinergic receptor activation, cathepsin release and lysosomal damage), the up-regulation of mitochondrial reactive oxygen species (ROS), the following release of oxidized DNA from mitochondria and the cardiolipin externalization may induce NLRP3 inflammasome activation [6,7]. Studies in macrophages and animal models have shown that oxidized low-density lipoprotein and cholesterol crystals trigger NLRP3 inflammasome activation [8,9]. In macrophage and animal models of uric acid accumulation, monosodium urate crystals activate the NLRP3 inflammasome, causing gout [10,11]. In addition, it is reported by Hecker and colleagues [12] that the activation of nicotinic acetylcholine receptors, containing subunits α7, α9 and/or α10, inhibited ATP-mediated IL-1β release by human and rat monocytes, helping protect them from collateral damage. NLRP3 inflammasome-related proteins are up-regulated in myocardial fibroblasts following infarction, and this up-regulation may contribute to infarct size in ischemia–reperfusion injury [13]. Caspase-1 maturation also supports, independently from IL-1β activation, pyroptosis, an important protection mechanism versus microbial infections. It stops pathogen replication inside the cell, promotes the phagocytosis of remaining bacteria and induces the release of pro-inflammatory proteins involved in the pathogenesis of different inflammatory chronic diseases [14,15]. Beside the canonical activation, a non-canonical one has been described. It depends on caspase-5 and caspase-4 in humans and on caspase-11 in mice [6]. In this model, in the first phase, Gram-negative bacteria (such as *Escherichia coli, Citrobacter rodentium, Legionella pneumophila, Vibrio cholerae* and *Salmonella typhimurium*) trigger the TLR4–MyD88 and toll/IL-1 receptor homology-domain-containing adapter-inducing interferon-β (TRIF) pathways, leading to the transcription of IL-18, IL-1β, NLRP3 and interferon regulatory factor (IRF)-7 and IRF3 genes via NFkB induction. The IRF7–IRF3 complex induces the expression of interferon (IFN)-α/β, leading to the activation of the janus kinase/signal transducers and activators of the transcription (JAK/STAT) pathway and caspase-11 gene transcription. In the second phase, unidentified receptors or scaffold proteins, activated by Gram-negative bacteria, cleave caspase-11. It induces IL-1α release, pyroptosis and IL-1β release through the canonical NLRP3 pathway [16]. Recently, a different non-canonical NLRP3 activation pathway, dependent on caspase-8, has been characterized [17,18,19,20,21]. Damage-associated molecular pattern molecules (DAMPs) and/or pathogen-associated molecular pattern molecules (PAMPs) may stimulate TLR4, leading to caspase-8 activation and its receptor. Receptor-interacting protein 1 (RIP1)–fatty acid synthase and (FAS)-associated death domain (FADD) protein may induce both canonical NLRP3 activation and the transcription step [21]. Misfolded protein amasses and an aberrant increase in several metabolites characterizing those disorders are endogenous DAMPs that have been demonstrated to be direct priming factors of the NLRP3 inflammasome. Additionally, mycobacteria and fungal cell wall component β-glucans, through dectin-1 stimulation, can induce the activation of a mucosa-associated lymphoid tissue lymphoma translocation protein 1 (MALT1)–caspase-8–ASC complex, which, in turn, contributes to IL-1β release [19]. Different from caspase-1, caspase-8 is as a direct IL-1β-converting protein. The NLRP3 inflammasome is expressed in many cells and is well characterized in macrophages, mainly, and neutrophils, monocytes and dendritic cells [2,22]. However, it is expressed in a huge variety of non-immune cells, but what an innate platform does in endothelial, hepatocytes and vascular smooth muscle cells is completely unknown [23]. Bone marrow-derived dendritic cells (BMDCs) lacking NLRP3 and ASC gene expression do not show caspase-8 activation and IL-1β processing [17,20]. Moreover, dendritic cell stimulation by fungal infection shows IL-1β release via the caspase-8–ASC association [19]. Overall, the activation of the NLRP3 inflammasome can be managed by different molecular processes, which may occur independently or be closely interconnected.

## 2. NRLP3 Inflammasome in Diseases

**Figure 2:**
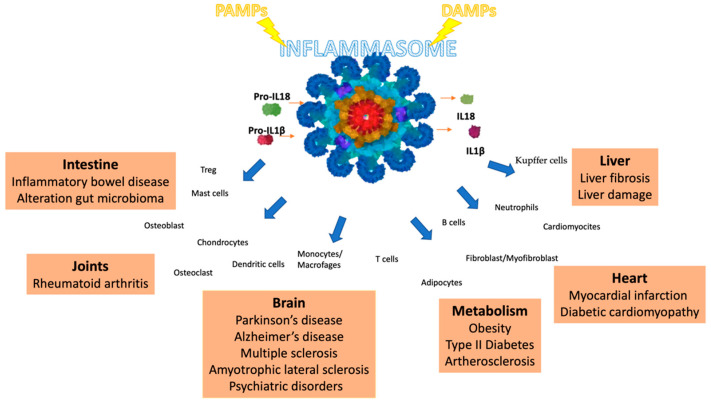
An overall summary of the NLRP3 inflammasome in pathologies.

While the innate immune response to invasions can efficiently protect against disease and death, the inappropriate activation of the NLRP3 inflammasome can contribute to the onset and progression of various diseases, as shown in Figure 2 [24,25,26,27,28]. The increased production of IL-1β and IL-18 by the NLRP3 inflammasome contributes to atherosclerotic plaque progression and instability in atherosclerotic patients and animal models [29]. 

In macrophage and animal models of type 2 diabetes and hyperglycemia, free fatty acids trigger inflammasome activation, which harms glucose metabolism and strengthens insulin resistance [30]. Taken together, these findings suggest that, during the progression of many metabolic diseases, the accumulation of abnormal metabolic products activates the NLRP3 inflammasome. 

Studies in animal models suggest a similar picture in Alzheimer’s disease [31] and obesity induced by a high-fat diet [32].

In macrophages and in animal models, studies have also defined a role for the NLRP3 inflammasome in the initiation and development of cerebral and myocardial ischemic diseases, including cerebral ischemia/stroke and myocardial ischemia [13,33]. Inflammasome activation appears to contribute to post-ischemic inflammation after stroke.

NLRP3 inflammasome activation has also been linked to various auto-immune and auto-inflammatory diseases. The severity of multiple sclerosis in patients correlates closely with levels of IL-1β, IL-18 and caspase-1 [34]. The serum levels of both ILs and of active caspase-1(p20) are elevated in mice with EAE [35]. Studies in macrophages and mouse models of colitis have linked abnormal NLRP3 inflammasome activation with inflammatory bowel disease, including ulcerative colitis and Crohn’s disease [36]. Polymorphism in the NLRP3 gene is linked to colitis severity and progression in patients [37], and gain-of-function mutations in the NLRP3 gene that increase the production and secretion of IL-1β and IL-18.

The new understanding of how inflammasomes are activated in health and disease raises new questions. As the mechanistic insight of the inflammasomes increases, opportunities to create new therapies for patients with inflammatory diseases are expected to enhance proportionately. In this review, we present the current knowledge regarding those pathologies which have inflammasomes among the main causes.

## 3. NRLP3 Inflammasome and Obesity

Obesity results in the disproportionate expansion of adipose tissue induced by immune cell infiltration and adipocyte hypertrophy [38]. Obesity is also characterized by the chronic inflammation of the adipocytes, leading to ectopic lipid accumulation and elevated levels of free fatty acids (FFAs) [39]. These conditions influence the development of several metabolic disorders, such as type 2 diabetes (T2D) and atherosclerosis. In obese patients, NLRP3 and ASC expression have been found to be up-regulated in the adipocytes [40]. In both mouse and human adipocytes during cellular differentiation and obesity development, caspase-1 levels are found to be increased [41]. In vitro experiments show that, when blocking caspase-1 and IL-1β there is an increase in adipogenic gene expression, indicating that caspase-1 is involved in adipogenesis via IL-1β. According to these data, caspase-1 inhibition may represent a novel therapeutic target in clinical conditions associated with obesity and insulin resistance because its deletion would inhibit the genesis of obesity [41]. Differentiated adipocytes lacking caspase-1 expression display better insulin sensitivity and adipogenesis than control cells [41]. In order to evaluate the connection between the development of obesity and inflammasome activity, several in vivo experiments were conducted. Genetically induced or high-fat diet (HFD)-induced obese mice with inflammasome component deficiency were studied [41,42]. Casp-1-/- mice show decreased fat mass, decreased adipocyte size, enhanced adipogenic gene expression and ameliorated insulin sensitivity. Moreover, these mice, when fed with HFD, increased in weight to a lesser extent than the controls. In genetically induced obese mice (ob/ob animals), caspase-1 blockade decreased mouse body weight. Additionally, caspase-1 inhibition led to decreased higher fat oxidation and lipogenesis than in the wild-type control animals without food intake [41]. 

In animal models of HFD-induced obesity, NLRP3, ASC and caspase-1 inhibition also ameliorated the disease [42]. Conversely, different studies observed the contradictory results. Casp-1-/- mice had more adipose tissue than the controls [43]. One of the possible reasons of these discrepancies may be the differences in intestinal microbiota in animals bred in different facilities, because the significant role of the intestinal microbiota in metabolic diseases has been demonstrated [44]. Moreover, mice with IL-18 deficiency were shown to have developed obesity and increased food intake compared to the controls [45]. This offers another possibility for the contradictory results in the obesity phenotypes detected in Casp-1-/- mice. Different mechanisms have been proposed for the role of caspase-1 in obesity. Previously, it has been suggested that macrophages migrate into the adipose tissue to produce caspase-1 [46]. Subsequently, in vivo studies have performed, which indicate that infiltrating macrophages are not involved in the major part of caspase-1 in adipose tissue [41]. Moreover, in non-hematopoietic cells, caspase-1 prevents lipid clearing through an NLRP3-dependent, but IL-18- and IL-1α/β-independent, mechanism [47]. Sirtuin 1 (SIRT1), a deacetylase that protects from obesity and regulates metabolism, has been identified as caspase-1 substrate. In HFD, caspase-1 cleaves an inactivated SIRT1 protein in adipose tissues, and the adipocytes in the absence of the SIRT1 gene show spontaneous obesity [48]. However, in adipocytes, the mechanism of caspase-1 and inflammasome activation needs clarification. In light of the fact that obesity is an ever-increasing pathology, more detailed studies aimed at identifying the roles and mechanisms of the action of NLRP3, or the clinical development of molecules to selectively antagonize the NLRP3 inflammasome, are necessary for a better understanding of the pathogenesis of comorbidities connected with obesity, as well as the development of pioneering approaches in precision medicine for the management of obesity and its complications.

## 4. NRLP3 Inflammasome and Type 2 Diabetes

Type 2 diabetes (T2D) is one of the major health issues producing insulin resistance. It results in elevated circulating levels of interleukins, TNF and adipokines [38]. In particular, IL-1β is involved in the pathogenesis of T2D, leading to the induction of insulin resistance and the promotion of β-cell impairment. In vitro experiments display that IL-1β reduces insulin sensitivity by promoting the phosphorylation of insulin receptor substrate-1 (IRS-1), leading to the down-regulation of the PI3K-Akt signaling induced by insulin. Additionally, it increases TNF-α expression [49], which, in turn, down-regulates insulin signaling [50]. IL-1β and the elevated FFAs increase oxidative and ER stress, which are involved in T2D pathogenesis [30,51]. Clinical analysis displays that an anti-IL-1β antibody and IL-1 receptor antagonist (IL-1RA) ameliorated β-cell function and glucose control levels [52,53]. Recent evidence demonstrates that NLRP3 activation contributes to diabetes-associated vascular dysfunction and pro-inflammatory phenotypes. In particular, diabetics (db/db) treated with a NLRP3 selective inhibitor (MCC950) showed reduced glucose levels, but body weight was not affected in db/db [54]. T2D patients show increased NLRP3 inflammasome activity in myeloid cells, compared to healthy people [55]. Mice lacking inflammasome components fed with HFD displayed improved insulin sensitivity and glucose tolerance [42,49,56,57,58]. They also show decreased inflammatory cytokine expression in metabolic tissues (adipose tissue and liver), serum and up-regulated insulin-PI3K-Akt signaling [42,49,56,57]. The reported studies offer a direct connection among chronic inflammation, NLRP3 inflammasomes and insulin resistance. Regarding the role of the NLRP3 inflammasome in the development of the T2D pathogenesis, several investigations have identified exogenous and endogenous stimulators of the inflammasome during T2D. It has described β-cells along with insulin secreting a peptide hormone called islet amyloid polypeptide (IAPP). It forms an amyloid structure in the pancreatic islets of T2D patients [50]. The CD36 receptor, the same involved in the transformation of oxidized low-density lipoprotein (oxLDL) into cholesterol crystals, converts soluble IAPP into its amyloid form. In cell cultures, IAPP stimulates NLRP3 activation, causing phagolysosome perturbation, as cathepsin-L and cathepsin-B induce IL-1β expression in the dendritic cells and macrophages [59]. In vivo studies show that, in animals with mouse β-cells that overexpress human IAPP, a strong induction of IL-1β has been detected in pancreatic macrophages [59,60]. Increased blood glucose has been shown to increment IL-1β levels in β-cells, probably by a mechanism which involves the thioredoxin-interacting protein (TXNIP) that, in turn, activates the inflammasome [57,61]. In particular, once glucose has up-regulated TXNIP expression in pancreatic islets, its combination with increased oxidative stress produces changes in the TXNIP structure, producing its dissociation from thioredoxin and association with the NLRP3 inflammasome [60]. These findings propose a link among NLRP3 activation, oxidative stress and IL-1β expression in islets. However, these data have not been confirmed in Txnip-/- macrophages [59]. The endocannabinoids and neuromodulatory lipids have been described as inductors of the IL-1β production inflammasome, dependent on pancreatic macrophages. This mechanism is mediated by the CB1 receptor (CB1R) and produces, in a paracrine manner, pancreatic β-cell death [62]. Anandamide, a neurotransmitter endocannabinoid, increases ASC expression and caspase-1 activation in islets and enhances IL-1β release from the macrophage cell line, RAW264.7. The IL-1β secretion induced by anandamide is related to Nlrp3 and Cb1r. In vivo studies have been conducted on Zucker diabetic fatty rats. They have a mutation of the leptin receptor gene, which leads to age-related hyperglycemia and reduced β-cell apoptosis. These data propose CB1R as therapeutic target in T2D [62]. Additionally, ceramide and palmitate that arise from HFD and promote T2D may enhance NLRP3 inflammasome activation [49,56]. In vitro studies show that palmitate can reduce AMP-activated protein kinase (AMPK) activity, producing the generation of mitochondrial ROS and defective autophagy that, in turn, activate the inflammasome [49]. Ceramide activates caspase-1 in epididymal adipose tissue and bone marrow-derived macrophages [56]. Moreover, changing saturated fatty acids with those that are monounsaturated in mouse HFD has been verified to reduce IL-1β expression through the AMPK pathway and improve insulin sensitivity [63]. In a mouse model of T2D induced by HFD, omega-3 fatty acids (ω-3 FAs) reduce insulin resistance, proposing its dietary use to ameliorate inflammatory diseases and T2D [64]. 

Despite increasing evidence of the relationship between the NLRP3 inflammasome, mitochondrial dysfunction and oxidative stress, and of their participation in type 2 diabetes physiopathology, therapeutic strategies to combat type 2 diabete, which target NLRP3 inflammasome, are yet to be consolidated. To date, the most used drug for the treatment of T2D is glibenclamide, capable of blocking the activation of the NLRP3 inflammasome [65]. However, further studies are needed to fully elucidate the specific mechanisms of the activation and regulation of the NLRP3 inflammasome in T2D.

## 5. NRLP3 Inflammasome and Atherosclerosis

Chronic inflammation has a key role in the beginning and development of metabolic disorders, such as obesity, type 2 diabetes (T2D), atherosclerosis and gouty arthritis [51]. In total, 70% of T2D patients develop atherosclerosis as a co-morbidity. The imbalanced lipid metabolism produces a progressive restricting of arterial vessels. In particular, white blood cells and cholesterol crystals amass in the vessels and reduce the oxygen flow to the organs [66]. Atherosclerosis is usually indicated as a furring or hardening of the arterial vessels and can cause life-threatening complications, such as stroke and heart attack. Several in vivo models [8,67,68] propose that IL-18, as a result of the inflammasome pathway, may have important role in this pathology. Additionally, the increased expression of IL-18 and its receptors has been found in human atherosclerotic plaques, compared to healthy arterial tissues. Based on these results, the attention has been focused on cholesterol metabolism and, in particular, on the apolipoprotein E (ApoE), fundamental for its proper metabolism. Elevated IL-18 levels have been found in ApoE-/- mice, which spontaneously occur in atherosclerotic lesions. This increased expression is related to the increased instability of atherosclerotic plaques and enhanced vascular inflammation. IL-18 deficient mice show smaller atherosclerotic lesion sizes [67,69,70]. Imbalanced lipid metabolism also produces the elevation of FFAs and LDL in patients’ blood, leading to increased pro-IL-1β expression via the TLRs, offering one of the first indicators of inflammasome activation [71]. CD36, a cell surface receptor, accelerates the cellular internalization of ox-LDL and its conversion in cholesterol crystals [72]. In vitro studies support the hypothesis that cholesterol crystals may activate the NLRP3 inflammasome through a mechanism involving cathepsin L88 and cathepsin B. In vivo evidences report that cholesterol crystals injected intraperitoneally produce acute inflammation that may be reduced by the absence of the NLRP3 inflammasome components, cathepsin L and cathepsin B. In this study, IL-1β is activated by the NLRP3 inflammasome pathway and promotes the breaking of the atherosclerotic plaques. When animals lacking the LDL receptor, already inclined to rising atherosclerotic plaques, are fed with a HFD, they show smaller lesion size if they also lack Asc, Nlrp3, IL-1 β or IL-1α [8]. In the same way, ApoE-/- mice subjected to atherosclerosis, showed a lack of IL-1β smaller lesions [73]. A different study displays that ApoE-/- mice subjected to atherosclerosis show decreased lesion sites when IL-1β is blocked [74]. According to other reports IL-1β and NLRP3 are not involved in atherosclerosis development, while IL-1α has a key role [75,76]. More investigations are necessary to elucidate the role of IL-1β and IL-1α in atherogenesis. Recently, intervention studies have been performed on smooth muscle cell (SMC) lineage, tracing ApoE−/− mice with advanced atherosclerosis using anti-IL-1β or IgG control antibodies [77]. Between 18 and 26 weeks of Western diet feeding, IL-1β antibody treatment resulted in a significant reduction in collagen content and SMC, but an up-regulated macrophage number in the fibrous cap. Additionally, no change was found in lesion size. SMC-specific Il1r1 KO led to smaller lesions nearly devoid of SMC and a fibrous cap, whereas the macrophage-selective loss of IL-1R1 had no effect on lesion size or composition. In this study, Gomez and colleagues show that IL-1β promotes multiple beneficial changes in late-stage murine atherosclerosis, including promoting the outward remodeling, formation and maintenance of a SMC/collagen-rich fibrous cap. Data reported by Bruder-Nascimento and colleagues show that the activation of IL-1R is also critically involved in the deleterious vascular effects of aldosterone [78]. Mice lacking the IL-1R or the inflammasome components NLRP3 and caspase-1 are protected from aldosterone-induced vascular damage. In addition, in vitro analyses show that aldosterone stimulates NLRP3-dependent interleukin-1β secretion by bone marrow-derived macrophages by activating nuclear factor-κB signaling and reactive oxygen species generation. Moreover, aldosterone increases the expression of NLRP3, active caspase-1, and mature interleukin-1β in human peripheral blood mononuclear cells. These data place NLRP3 as a potential target for therapeutic interventions in conditions with high aldosterone levels.

We can conclude that the modern hypotheses introduce atherosclerosis as an inflammatory/lipid-based disease and the NLRP3 inflammasome has been considered as a link between lipid metabolism and inflammation because crystalline cholesterol and oxidized low-density lipoprotein (oxLDL) (two abundant components in atherosclerotic plaques) activate the NLRP3 inflammasome.

## 6. NRLP3 Inflammasome and Ischemic Heart Disease

Myocardial infarction (MI) occurs when blood flow stops or decreases in a part of the heart, leading to heart tissue damage. Usually, reperfusion therapy is the most employed in the treatment of this disease. Reperfusion is often accompanied by inflammation, which is indispensable in scar formation and wound healing, but may also produce adverse remodeling [79]. Cardiac tissues from patients subjected to MI show infiltrated cells, in particular, macrophages and neutrophils with increased ASC expression [80]. Moreover, when subjected to ischemia/reperfusion (I/R) injury, animals lacking ASC gene expression show reduced inflammatory cell infiltration, smaller infarcted area and improved cardiac remodeling, as compared to the wild-type mice. In vitro evidence shows that, six hours after LPS stimulation, no inflammatory response was activated in the cardiomyocytes, while it was detected in cardiac fibroblasts, suggesting that it may involve potassium efflux and cellular ROS generation [80]. Clinical reports show that patients with MI display elevated expression of the NLRP3 inflammasome components [81]. The same trend has been reported in in vivo experiments [13]. In particular, experiments conducted ex vivo show that the hearts of NLRP3−/− mice exposed to I/R injury have preserved heart function, as compared to the ASC−/− heart [13]. Contradictory results show that, 24 h after I/R injury, there was a larger infarct size in animals lacking NLRP3 gene expression, while no difference was detected in lymphocyte infiltration [82]. When NLRP3 −/− and ASC−/− mice were administered with a cardioprotective stuff before I/R, no positive outcome was detected, proposing the protective role of the NLRP3 inflammasome in I/R [82]. These discordant results might be explained by considering the different timepoints used in these studies, because at the early stage, the inflammasome was not activated [83,84]. In a MI model when NLRP3 inhibitors were administered to wild-type animals, no changes in infarct size were detected three hours after reperfusion, as compared to the non-administered animals, while smaller infarct size was shown at 24 h. Moreover, these positive effects were observed when the inhibitor was administered one hour after reperfusion or immediately, while no beneficial effects were shown when the inhibitor was administered three hours after reperfusion, demonstrating that the inhibition of the NLRP3 inflammasome has a narrow therapeutic window [84]. As a product of the NLRP3 inflammasome complex activation, caspase-1 displayed negative effects on MI by activating pyroptosis [85]. Recent evidence shows that, in addition to fibroblasts, cardiac microvascular endothelial cells are also involved in the inflammatory response induced by I/R [86]. In vivo experiments, showing that TXINP siRNA manages NLRP3 inflammasome activation, suggest that TXINP mediates its activation in endothelial cells. 

To date, we have deduced that the NLRP3 inflammasome contributes significantly to the pathological process of ischemic heart disease. However, outcomes from experiments manipulating the NLRP3 inflammasome in myocardial ischemia remain indefinite. In conclusion, the targeting the NLRP inflammasome in cardiovascular disease treatment holds promise, and the optimization of therapeutic approaches requires further clarification regarding the precise role of NLRP3 in cardiovascular disease.

## 7. NRLP3 Inflammasome and Non-Ischemic Heart Disease

Diabetic cardiomyopathy (DCM) is a complication of diabetes usually observed at the terminal stage of the disease. It has been described that the inflammasome has a key role in this process. In particular, glucose has been identified as one of the main inducers of the NLRP3 complex [87]. In diabetic rats, NLRP3 gene silencing shows reduced fibrosis, cardiac inflammation, cardiac dysfunction and cell pyroptosis [88]. Moreover, by inhibiting ROS production, an important mediator of inflammasome activation, the TXINP and NF-κB pathways may be abolished and reduce the proinflammatory cytokine release [88]. To further evaluate NLRP3 inflammasome activation, an in vivo model of chronic heart failure was prepared with the calcineurin transgene (CNTg), heterozygously overexpressed, specifically in the heart [89]. CNTg mice showed increased NLRP3 mRNA levels along with inflammation, cardiac hypertrophy and ventricular dilatation. In line with these results, treatment with the IL-1 receptor antagonist or NLRP3 gene deletion rescued systolic dysfunction and reduced cardiac inflammation [89]. Moreover, a fundamental role of the NLRP3 inflammasome was confirmed in hypertension [90]. In a transverse aortic constriction (TAC) mouse model, a widely use animal model of hypertension, NLRP3 inflammasome component expression was significantly increased while cardiac function was impaired [90]. In a sepsis animal model, an increase in cardiac fibroblasts pre-treated with lipopolysaccharide (LPS) was observed, as well as increased NLRP3 expression and myocardiac dysfunction [91]. The NLRP3 inflammasome, activated by K^+^ efflux and ROS increase, is also critical in the development of myocarditis in the coxsackievirus B3- (CVB3)-induced model [92]. Moreover, by deactivating NLRP3, the VEGF-A negative effects on myocardial function in an aged heart was partially abolished. Such a positive impact is obtained by disabling NLRP3, but not IL-18 or IL-1, suggesting that NLRP3 should regulate cardiac function, independent of the inflammasome [93]. 

Surprisingly, in the aged heart, a sustained increase in VEGF-A was found to exert negative effects on cardiac function. Such adverse impacts could be partially abolished by disabling NLRP3, but not IL-1R or IL-18, indicating that NLRP3 may participate in regulating cardiac function independent of the inflammasome.

Here we have reported the current information about the NLRP3 inflammasome’s contribution to non-ischemic DCM and reported several possible underlying mechanisms. To date, a lot of information is still lacking and further investigations are required. 

## 8. NRLP3 Inflammasome and Liver Diseases

Recent studies have shown that the inflammasome has an important role in the pathogenesis of liver disease. The components of the inflammasome have been found in various types of liver cells. For example, NLRP3 expression was observed in Kupffer cells, hepatocytes and sinusoidal endothelial cells [94]. In one study, it was shown that acetaminophen-induced hepatotoxicity was due to the involvement of the inflammasome. In fact, in the absence of NRLP3, there is a reduction in mortality in mice treated with acetaminophen [95,96]. The involvement of NLRP3, whose expression is observed mainly in hepatic stellate cells, has also been demonstrated in a mouse model of liver fibrosis [97]. Furthermore, an improvement in liver inflammation and the minor lesion of the tissue following ischemia reperfusion was observed in NLRP3 knockout mice [97]. Subsequent studies have shown that the inflammasome does not come into action only after tissue injury in sterile models of liver disease, but also following exposure to microorganisms or microbial components [98,99]. Recently, the involvement of the inflammasome in the development and progression of nonalcoholic steatohepatitis (NASH) has been shown. In fact, the upregulation of genes associated with the inflammasome, following the induction of the mouse model of NASH, has been observed in isolated hepatocytes. From the point of view of the mechanism of action, it has been discovered that, in response to LPS, palmitic acid activates the inflammasome in the hepatocytes where IL-1β is released. It has also been shown that the microbial and non-microbial ligands of the pattern-recognition receptor (PRR) act together to induce pathogenic inflammasome responses in the liver [100,101]. An interesting discovery was that NLRP3 can aggravate liver damage by acting on the intestinal flora in the intestine–liver axis. NLRP3 knockout mice, fed on a Western diet and with water containing fructose, presented an alteration of the intestinal antimicrobial peptide synthesis. This led to an increase in the permeability of the intestinal mucosa with consequent dysbiosis (increase in the Firmicutes/Bacteroidetes ratio) and an increase in proteobacteria. These events, in turn, led to bacterial translocation and the increased expressions of both the LPS receptor, TLR4, and the double-stranded bacterial DNA receptor, TLR9, in the liver [102].

Evidence from mouse models and early clinical studies in patients with non-alcoholic steatohepatitis and fibrosis supports the notion that the NRLP3 inflammasome can be successfully translated into novel treatment options for patients with liver disease.

## 9. NRLP3 Inflammasome and Inflammatory Bowel Diseases

Inflammatory Bowel Diseases (IBDs), such as ulcerative colitis (UC) and Crohn’s disease (CD), comprise relapsing and chronic inflammatory disorders that concern the gastrointestinal tract [103]. Several studies show that the NLRP3 inflammasome is involved in the maintenance of intestinal homeostasis and, during bowel inflammation, manages the innate immune responses, contributing to the support of the ongoing inflammation and the interruption of the enteric barrier through a modification of the tight junction proteins and cell apoptosis [6,104]. Clinical evidence reports that the IL-1β secretion from macrophages and colon tissues of patients affected by IBD increase with the severity of the disease [105]. NLRP3, ASC and IL-1β expression has been found to increase in the colon mucosa of IBD patients [106]. Several in vivo studies have investigated the effects of pharmacological inhibition or the gene deletion of the NLRP3 inflammasome components in colitis animal models [6]. It has been described that the absence of molecules involved in both non-canonical and canonical NLRP3 inflammasome activation has both detrimental and protective roles in IBDs, depending on the experimental model used. In particular, it has been proposed that, in the first step of enteric inflammation, it contributes to the maintenance of barrier integrity and tissue repair, while, when inflammation becomes chronic, its over-activation produces the excessive release of IL-18 and IL-1β that contribute to inflammatory/immune responses [6]. Mice lacking Nlrp3, Asc and caspase-1 gene expression are more vulnerable to experimental colitis induced by 2,4,6-trinitrobenzenesulfonic acid (TNBS) and dextran sodium sulfate (DSS), both characterized by diarrhea, body weight loss, rectal bleeding and mortality, proposing the favorable action of NLRP3 complex in the colon tissue. This protective action has been assigned to the capacity of NLRP3 of producing IL-18 release, which is a key mediator for repairing the mucosal barrier of the digestive tract. In addition, the absence of Nlrp3 gene expression leads to an increase in nitric oxide (NO) levels and a decrease in IL-10, an anti-inflammatory cytokine, and TGF-β expression [107]. NLRP3 activation is also able to manage leukocyte recruitment and the trafficking of neutrophils, modifying migration, chemotactic responses and acting as a reparative key. 

Different findings point out the hypothesis that a lack in NLRP3 inflammasome components may protect animals from DSS colitis [108,109]. Nlrp3-/- mice, treated with DSS, show reduced pro-inflammatory cytokine expression and develop less severe colitis, as compared with WT mice. Moreover, pralnacasan administration, a caspase-1 inhibitor, reduces colon tissue damage as does NLRP3 deficiency, proposing that NLRP3 inflammasome blockades may be a pharmacological strategy for the modulation of IBDs [109,110]. In support of this theory, recent in vivo evidence has displayed that drugs targeting different phases of NLRP3 activation, including defense against mitochondrial damage, the inhibition of NF-kB nuclear translocation, the reduction in pro-caspase-1 cleavage, the activation of the NFE-related factor 2 (Nrf2) pathway, the direct blockade of the IL-1β receptor or non-canonical and canonical NLRP3 activation, show positive effects on IBDs [111,112,113,114,115,116]. The different roles ascribed to the NLRP3 inflammasome in IBDs may be attributed to different experimental conditions. For example, in studies displaying the regulatory and protective action of NLRP3, experiments have been conducted on the seventh day, after two days without DSS treatment, five days with 3% DSS exposure, or seven days with 2.5% DSS treatment. By contrast, a detrimental role was ascribed to NLRP3 in mice administered for nine days with 2% DSS. It has been proposed that, by extending DSS administration, increased NLRP3 activation becomes detrimental for colon tissue [109]. 

Based on the above, the NLRP3 inflammasome acts as an important factor in the pathogenesis and progression of IBD. However, further experiments are needed to develop effective therapies. In particular, since the correct level of activation of the NLRP3 inflammasome is considered necessary in the maintenance of intestinal homeostasis [107,117], it is necessary to make further efforts in the search for the correct application of the NLRP3 inflammasome inhibitors in the treatment of IBD.

## 10. NRLP3 Inflammasome and Gut Microbiome

There are over 500 different types of bacteria in our intestine. Intestinal microflora play countless roles, including metabolic, immunological and protective functions [118,119]. Although the microbiome is not an immune component of the intestinal mucosa, it can interact with immune cells and soluble components contributing to the immunity of the mucosa [120,121]. Recent studies have highlighted the important role of the inflammasome on the regulation of the composition of the intestinal microflora in mouse models. In this research it was observed that the absence of components of the inflammasome was associated with dysbiosis, which can lead to a greater susceptibility to colitis and tumorigenesis. The factors implicated in the development of dysbiosis still remain to be well understood. In recent years, co-housing studies have been conducted between WT mice and ASC-/-, NLRP6-/- or caspase-1 sensitive mice. In all of these cases, it was observed that WT mice, co-housed with inflammasome-deficient mice, developed more severe colitis than those housed individually [122,123,124]. This has been explained with studies aimed at identifying bacteria in the different types of mice that showed an altered microbiome, characterized by the presence of a higher number of potentially colitogenic bacteria in mice deficient in inflammasomes and, in particular, in mice with NLRP6 deficiency, which can be transferred to WT mice after cohousing. In particular, Prevotella and the phylum TM7 have been identified in the NLRP6-/-, ASC-/-, caspase1-/- and IL-18-/- mice and in the cohoused WT mice, which developed severe colitis following treatment with DSS [122,125]. Other studies have shown an alteration of the composition and quantity of the microbiota, even in mice with NLRP1 and NLRP3 deficiency. In particular, the analysis of bacteria showed an enrichment of *Enterobacteriaceae*, a bacteria belonging to the *S. thuringiensis* family, including the different Clostridium, Rod bacteria and Proteobacteria [126,127].

How the microbiota present in the intestines of inflammasome-deficient mice promote dysbiosis remains to be clarified, but this may be linked to the ability of these bacteria to upregulate the production of proinflammatory mediators. From the studies carried out in recent years, it can be said that the lack of inflammation signaling leads to disturbances in the intestinal microbiota, which, in turn, entails an accumulation of bacteria capable of intensifying pro-inflammatory responses, predisposing to inflammation-related diseases, including colitis, tumorigenesis and metabolic syndrome [128,129,130]. Further support comes from recent research focused precisely on the crosstalk complex between the inflammasome NLRP3 and the intestinal microbiota. From this research, it has been discovered that the hyperactive inflammasome NLRP3 leads to a local overproduction of IL-1β. This phenomenon could maintain intestinal homeostasis and confer greater resistance to experimental colitis through a remodeled intestinal microbiota with an increased anti-inflammatory capacity. This capacity was, in turn, due to an increase in the induction capacity of regulatory T cells [131]. A microbiota inflammasome regulation mechanism studied in recent years is based on the IL-18/antimicrobial peptides (AMP) axis. In these studies, it was observed that the greatest susceptibility to the development of colitis in ASC-/-, NLRP6-/-, NLRP1-/-, NLRP3-/-, AIM2-/- or caspase-1-/- mice was related to a decrease in IL-18 levels [132]. Additionally, IL-18-/- mice have been shown to develop more severe DSS-induced colitis than WT mice [122]. IL-18 is capable not only of inducing Th1 responses through the upregulation of INFγ but also of upregulating the production of AMP, important for bacterial clearance. As with IL-18, AMP levels also decreased in inflammasome-deficient mice compared to WT mice and the administration of recombinant IL-18 brought AMP levels back to normal [124,133]. In detail, the NLRP6-/-, ASC-/- or IL-18-/- mice had decreased levels of AMPs, Ang1, Ang4, Relnβ and Itln1, while the AIM2-/- mice had low levels of Reg3γ, Reg3β, and β-defensin 2 [134,135]. Other studies have shown that the injection of Ang4 in ASC-/- mice led to changes in the diversity and structure of the intestinal microflora [124,136]. This supported the idea that the production of AMP can contribute to the enrichment of some bacterial populations in knockout mice for the components of the inflammasome. 

In conclusion, we can affirm that intestinal commensal microbes can stimulate excessive or persistent inflammation in genetically sensitive subjects, shedding light on the elucidation of the etiology of IBD (104). Furthermore, the reported information suggests the important role of the NLRP3 inflammasome on the regulation of the composition of the intestinal microflora and offers new therapeutic targets for the maintenance of intestinal homeostasis.

## 11. NRLP3 Inflammasome and Rheumatoid Arthritis

Rheumatoid Arthritis (RA) is an autoinflammatory disease characterized by irreversible joint destruction and synovial inflammation, producing motor impairment and premature mortality [137]. Polymorphisms of the NLRP3 gene are related to RA incidence and pathology severity [138,139,140]. Moreover, clinical reports show that inflammasome components (mRNA and protein expression) are increased in RA patient synovial fluids and circulating macrophages/monocytes, neutrophils and dendritic cells, proposing that NLRP3 activation is involved in both systemic and local inflammation in RA [141,142,143,144,145]. However, current evidence does not explain the exact mechanism by which the NLRP3 inflammasome in involved in the pathophysiology of RA, or whether it is activated as consequence of the synovial inflammatory process. To clarify this point, several in vivo experiments were conducted. Animals lacking IL-18 gene expression were less vulnerable to the arthritis induced by collagen (CIA), as compared with the control, suggesting the key role of the IL-18 cytokine in RA [146]. In another paper, CIA mice showed increased NLRP3, caspase-1 and IL-1β expression in sera and knee joint synovia [147]. In addition, the same authors showed that MCC950, a selective NLRP3 inhibitor, by decreasing IL-1β production, reduces bone destruction and joint inflammation. They suggest that NLRP3-induced IL-1β activation contributes to sustaining and shaping the inflammatory/immune processes in RA, thus, the NLRP3 inflammasome may be a suitable pharmacological target of RA treatment [147]. Furthermore, further research has shown increased NLRP3 activity in the peripheral blood cells of RA patients. In particular, these patients had higher basal intracellular levels of NLRP3, ASC, caspase-1 and pro- IL-1β [142]. This finding suggests that targeting NLRP3 or downstream caspases can have beneficial effects on suppressing IL-1 production in RA. For the moment, this is the most studied and most effective anakinra for the treatment of RA [148,149]. Another important discovery, recently made, is that, in mice with specific deletion in the myeloid cells of the Tnfaip3/A20 gene, there was a development of spontaneous erosive polyarthritis, similar to RA in patients. This polyarthritis is linked to the NLRP3 inflammasome and IL-1R signaling. As proof of this, there was a study done on myeloid cell-specific A20^-/-^ mice, in which an improvement of arthritis was observed in these animals following the deletion of NLRP3, caspase-1/11 or IL-1R. Therefore, thanks to this study, it can be stated that myeloid cell-specific A20^-/-^ mice can be used as a pre-clinical models for the validation of therapies for RA that have the aim of modifying inflammasome or IL-1 signaling [150].

The roles of the NLRP3 inflammasome in the development of RA have not yet been elucidated fully. New perspectives for the discovery of innovative drugs are linked to recent discoveries that highlight that the aberrant activities of inflammasomes cause pyroptosis, a lytic form of cell death that simultaneously triggers several inflammatory mediators in the extracellular environment. As a result, strategies are being formulated, aimed at preventing pyroptosis through the selective blocking of the single components of the inflammasomes or the inhibition of the signaling nodes that integrate numerous inflammatory signals, such as MAP38 p38.

## 12. NRLP3 Inflammasome and Parkinson’s Disease

Parkinson’s disease (PD) is characterized by the death of dopaminergic neurons in substantia nigra and the aggregation of α-synuclein (αSyn) in neurons [151]. αSyn can aggregate into fibrils with a characteristic β-sheet structure, very similar with the amyloid fibrils of the Alzheimer’s disease (AD) [152]. Moreover, with different intracellular mechanisms, αSyn may be released outside of the cell [16]. Once arrived in the extracellular space, αSyn induces the activation of astrocytes and microglia and increases the production of IL-1β [16,153]. Additionally, in human primary monocytes, both monomeric and fibrillary αSyn increase pro-IL-1β levels via TLR2 signaling, but only the fibrillary form of αSyn fully triggers the inflammasome by activating caspase-1 and producing the maturation of IL-1β [154]. In particular, cathepsin B, phagocytosis and ROS are required for caspase-1 activation. Thus, it has been supposed that ROS and cathepsin B are upstream of NLRP3 activation, proposing that αSyn promotes the activation of the NLRP3 inflammasome [154]. However, in this study, conducted by Codolo et al. (2013), the activation of NLRP3 has been described by an in vitro model [154]. In a PD animal model, induced by neurotoxin 1-methyl-4-phenyl-1,2,3,6-tetrahydropyridine (MPTP) injections, which induce the loss of dopaminergic neurons in substantia nigra, the Nlrp3 deficient mice were shown to be resistant to PD. Here, there is in vivo evidence for the involvement of the NLRP3 inflammasome in PD [155]. Moreover, it has been described that dopamine negatively regulates NLRP3 priming in both astrocytes and microglia by the dopamine D1 receptor (DRD1)-cyclic adenosine monophosphate (cAMP) signaling pathway [155]. Interestingly, cAMP binds NLRP3 and induces its degradation by the E3 ubiquitin ligase, MARCH7 [155]. Additionally, DRD1-/- mice are less resistant to neuroinflammation induced by MPTP injections, as shown by increased NLRP3 activation, IL-18 and IL-1β production and the enhanced reduction in dopaminergic neurons [155]. 

These studies propose that the NLRP3 inflammasome and dopamine-producing neurons may regulate each other. In particular, the inflammasome impaired these cells, while dopamine inhibited NLRP3 activation. The drugs currently in use (NSAIDs of fenamate and artemisinin of the antimalarial drug) have been shown to inhibit the inflammasome NLRP3 by protecting against AD [156,157]. However, these drugs seem to cause unwanted side effects. Therefore, studies are needed for the development of new treatments focused on the main mediators of the activation of the inflammasome.

## 13. NRLP3 Inflammasome and Alzheimer’s Disease

Alzheimer’s disease (AD) results in the accumulation of amyloid-β peptide, forming plaques, in the cerebral tissue. When AD occurs the amyloid-β peptide, normally produced in the cerebrum by the cleavage of the precursor protein, it forms prion-like misfolded oligomers [158]. Amyloid-β was the first peptide associated with neurodegenerative disease that was discovered to stimulate the mouse NLRP3 inflammasome, producing IL-1β activation [159]. Halle and colleagues show that, in LPS-primed mouse macrophages, fibrillary amyloid-β produces NLRP3 inflammasome-induced caspase-1 activation through a mechanism primed by cathepsin B release and endosomal rupture [159]. Moreover, cathepsin B inhibitor treatment reduced amyloid plaque load and improved memory deficit in an animal model of AD, proposing the inflammasome as therapeutic target in AD [160]. Recent studies propose that CD36, a cell-surface receptor, may promote the internalization of amyloid-β. It is intracellularly converted into its fibrillary form which, in turn, activates the NLRP3 inflammasome [72]. In transgenic mice that produce the chronic deposition of amyloid-β, called APP/PS1 mice, those lacking in NLRP3 and caspase-1 have been described as a link between the NLRP3 inflammasome pathway and AD. These animals displayed reduced AD-related comorbidities, reflected by decreased chronic amyloid-β secretion, cognitive impairment and neuronal inflammation. In these animals, the absence of the NLRP3 inflammasome drove the microglial cells towards an M2 phenotype, which is characterized by the increased expression of IL-4 and arginase-1. This change caused the decreased deposition of amyloid-β and increased tissue remodeling in mice affected by AD [161]. From a clinical point of view, caspase-1 expression has been found to be enhanced in the brains of patient with AD, proposing a link between AD in humans and inflammasome activation [161]. 

Therefore, in vitro and in vivo studies suggest a potentially important role for the NLRP3 inflammasome in the pathogenesis of AD and identify the NLRP3–caspase-1 axis as a potential target for AD therapy. Currently, the application of inhibitors targeted at NLRP3 is limited due to a lack of in-depth understanding. Furthermore, interfering with the main inflammasome mediators (NLRP3, ASC and Caspase-1) can have serious side effects, considering that the activation of the inflammasome is omnipresent [162]. Further studies are needed for the development of new treatments that could focus on the specific pathways of NLRP3 activation in the CNS.

## 14. NRLP3 Inflammasome and Multiple Sclerosis

Multiple sclerosis (MS) is one of the most widespread autoimmune inflammatory disorders. It is characterized by infiltrate myelin-reactive CD4+ T cells in the central nervous system (CNS). Moreover, it induces demyelination it and attacks oligodendrocytes [163]. The induced demyelination disrupts the communication of the CNS and peripheral nervous system (PNS) and causes mental, physical and psychiatric issues. Nowadays, MS has no resolutive cure and is responsible of a reduction in the life expectancy of patients by 5–10 years [164]. The most employed animal model to mimic MS is the experimental autoimmune encephalomyelitis (EAE) mouse model. Animals are injected with the myelin oligodendrocyte glycoprotein peptide (MOG) dissolved in adjuvant. This immunization induces the recruitment of inflammatory cell types, including MOG-specific T cells, into the nervous system [165]. Before the discovery of the inflammasome pathways, its products, caspase-1, IL-18 and IL-1β, were already described as being involved in EAE progression. Mice lacking caspase-1, IL-1β, IL-1α and IL-18 gene expressions are immune to EAE induction and display a concomitant down-regulation in IL-17 and/or interferon (IFN)-γ levels [166,167,168]. Recent evidence shows that, in the spinal cord, Nlrp3 expression is increased during EAE, while Nlrp3-/- mice have a delayed course and reduced severity of pathology, with less astrogliosis and reduced infiltrating inflammatory cells [165,169]. Additionally, in another experimental model of MS Nlrp3-/-, animals have lower oligodendrocyte loss and demyelination [170]. Moreover, mice subjected to EAE display increased IL-18 expression than the controls and mice lacking IL-18 expression have the same delayed disease progression observed in the Nlrp3-/- mice, proposing that the Nlrp3 pathway, through IL-18 expression, may promote EAE [165,169]. However, in EAE progression, the role of NLRP3 is still intricated. In the antigen-presenting cells (APCs), Nlrp3 expression is mandatory to stimulate T helper type 17 (TH17) and TH1 cells to answer to brain autoantigens [165]. It has been described that Nlrp3 and Asc absences produce a down-regulated expression of several chemokines and chemokine receptors, such as Ccr6 and Ccr2 in TH and APCs cells, decreasing the migration of the TH17 and TH1 cells into CNS in the Nlrp3-/- and Asc-/- mice, following MOG injection. These findings suggest that the NLRP3 inflammasome pathway is involved in TH17 and TH1 cell migration and response during EAE. From a clinical point of view, patients with relapsing MS have been found to have peripheral blood mononuclear cells (PBMCs) with higher expressions of NLRP3, caspase-1 and IL-1β compared to the healthy controls. To support the link between the NLRP3 inflammasome and MS, it has been proposed that, upon NLRP3 activation, human PBMCs secrete soluble factors that move the cytokine profile towards a TH17 phenotype [171]. The role of the NLRP3 inflammasome in EAE is not confirmed in all studies and changes according to the disease model. EAE induction by heat-killed mycobacteria (Mtb), a more aggressive immunization method, also affected Nlrp3-/- and Asc-/- mice. However, NLRP3 and ASC are required when the Mtb immunization dose is lower [35]. In another study conducted by Shaw and colleagues, both WT and Nlrp3-/- mice developed EAE after MOG immunization. They propose that EAE progression is promoted by ASC through an inflammasome-dependent pathway, which contributes to CD4+ T cell survival. In the same study, they showed that Asc-/- mice were more resistant to EAE induction compared to Casp1-/- mice [172]. To explain these differences in inflammasome involvement, it was proposed that IL-1β activation by macrophages may be inhibited by IFN-β, showing that IFN-β treatment reduced only NLRP3-dependent EAE. These findings propose that IFN-β may therapeutically inhibit the NLRP3 inflammasome, IL-18 and IL-1β in MS [35]. However, this disease is really heterogenous. In fact, even if IFN-β has been use in therapy for more than 20 years, only two-thirds of MS patients respond to its therapy. Using another experimental model of EAE, involving induction by pertussis toxin (PTX) injection, it has been shown that, for IL-1β induction, TLR4 is required and the pyrin-dependent inflammasome is involved in pro-IL-1β induction. Bioactive IL-1β stimulates stromal cells to produce IL-6, which, in turn, activates leukocyte migration and adhesion. Mice with transgenic MOG-specific T cell receptors, known as Pyrin-/- 2D2 mice, showed fewer EAE incidences and less severe and delayed pathology following PTX injection. Besides, the pyrin inflammasome works only at the early stage of PTX-induced EAE, as the equal migration of CD3+ cells have been detected in the spinal cord of animals with the same clinical score independent from the genotype. In line with this finding, the adoptive injection of MOG-specific T cells into pyrin-/- and WT mice produced similar EAE [173]. 

In conclusion, this evidence supports the importance of the NLRP3 inflammasome not only in the pathogenesis of EAE, but also in the different stages of disease development, such as initial inflammation, T-cell inclination, breakage of the CNS barrier and neurodegeneration. This highlights the need to identify the locations and cellular sources of inflammasomes and IL-1β in EAE and MS to fully understand their impact on the diseases.

## 15. NRLP3 Inflammasome and Amyotrophic Lateral Sclerosis

Amyotrophic Lateral Sclerosis (ALS) is a neurodegenerative disease that affects upper and lower motor neurons. Patients with this condition usually die from neurogenic muscle weakness. An increase in IL-18 levels has been observed in the sera of ALS patients, suggesting that the inflammasome has a key role in the development of this disease [174]. To support this hypothesis, there are studies showing an increase in the expression of NLRP3 and Casp-1 in the brain tissues of patients with ALS [175]. High levels of genes associated with the inflammasome have also been found in the post-mortem tissue of patients with ALS. Some researchers recently conducted a study on the spinal cord to evaluate the inflammasome complex. This study showed that the cells of the spinal cord that express the most NLRP3 components are astrocytes [176]. The implication of the inflammasome in this pathology offers a new therapeutic target on which to act. Recently, 17β-estradiol was shown to improve motor neuron survival in a humanized animal model of ALS that carried the human SOD1 (G93A) mutation. This result is due precisely to the ability of this steroid hormone to down-regulate the activation of the inflammasome [177].

Taken together, these findings demonstrate that the brain tissues of ALS patients express NLRP3, and that pathological ALS proteins activate the NLRP3 inflammasome. NLRP3 inhibition may, therefore, be a potential therapeutic approach to arrest microglial neuroinflammation and ALS disease progression.

## 16. Other Neurological Disorders

Rasmussen’s Encephalitis (RE) is an inflammatory encephalopathy characterized by convulsions and progressive cognitive disabilities. Cortex and white matter samples taken from RE patients had high levels of IL-1β, IL-18, NLRP1, NLRP3 and Casp-1 [178].

HIV-1 infected patients may also have neurological changes [179]. Neuropathologies in patients with HIV-1 have been associated with the activation of perivascular macrophages, microglia residing in the brain and the consequent release of proinflammatory cytokines [180]. Subsequent studies have shown that the increase in these cytokines was linked to the activation of the inflammasome. In fact, NLRP3 deficiency inhibited the translocation of ASC and the activation of Casp-1 [181,182]. In addition, a protein that is released from HIV-1 infected cells, called viral protein R, is capable of inducing the splitting of Casp-1 and the consequent release of IL-1β into macrophages, dependent on NRLP3 [183].

Another neurological disorder is represented by prion disease, a neurodegenerative and neuroinflammatory disorder due to the accumulation of the prion protein. This accumulation leads to the activation of the microglial and the release of proinflammatory cytokines. Additionally, in this case it has been shown that the inflammasome has a fundamental role in the pathology [184,185].

Our data also suggest that further elucidation of the inflammasome–brain axis may offer novel therapeutic targets for psychiatric disorders. 

## 17. Conclusions

The inflammasome plays an important role in various inflammatory, metabolic, neurological and cardiovascular diseases. Therefore, it represents a promising target for the treatment of various pathologies. To be used as a therapeutic target, however, it is important to understand the balance between the activation of the beneficial and harmful inflammasomes. In particular, researchers need to aim at a mechanistic understanding of inflammasomes so that new therapies can be created for patients with these diseases.

To date, there are still numerous questions which researchers must answer. For example: Is it possible to modulate the activation of the inflammasome by post-translational modifications of the inflammasome? What is the contributing role of the inflammasome in myeloid cells or other types of cells (epithelial, endothelial, etc.) during inflammation? Will it be possible to identify drugs capable of directly affecting the components of the inflammasome rather than its final products, such as IL-1β?

Despite the fact that several NLRP3 inflammasome inhibitors have been developed, further basic and clinical research is needed for a better understanding of inflammasomes and for the development of effective therapeutic strategies for both the prevention and treatment of inflammatory, metabolic, neurological and cardiovascular diseases, which have inflammasomes among the main causes.

## Abbreviations

α -syn	α-synuclein
Aβ	β-amyloid
AD	Alzheimer’s disease
AIM2	absent in melanoma 2
ALS	amyotrophic lateral sclerosis
AMP	antimicrobial peptides
APCs	antigen-presenting cells
APOE	apolipoprotein E
ASC	apoptosis-associated speck-like protein containing a carboxy-terminal CARD
BMDCs	bone marrow-derived dendritic cells
CARD	caspase activation and recruitment domain
CD	Crohn’s disease
CIA	arthritis induced by collagen
CNS	central nervous system
DAMP	danger-associated molecular pattern
DCM	diabetic cardiomyopathy
DSS	dextran sodium sulfate
EAE	experimental autoimmune encephalomyelitis
FFAs	free fatty acids
HFD	high-fat diet
IAPP	islet amyloid polypeptide
IBDs	Inflammatory Bowel Diseases
IFN	interferon
IL	interleukin
IL-1R	IL-1 receptor
IL-1RA	IL-1R antagonist
IRF	interferon regulatory factor
IRS-1	insulin receptor substrate-1
LDL	low-density lipoprotein
LPS	lipopolysaccharide
LRR	leucine-rich repeat
MALT-1	mucosa-associated lymphoid tissue lymphoma translocation protein 1
MCP-1	monocyte chemoattractant protein-1
MI	myocardial infarction
MOG	myelin oligodendrocyte glycoprotein peptide
MPTP	1-methyl-4-phenyl-1,2,3,6-tetrahydropyridine
MS	multiple sclerosis
MyD88	myeloid differentiation primary response 88
NADPH	nicotinamide adenine dinucleotide phosphate
NF-kB	nuclear factor kappa-light-chain-enhancer of activated B cells
NLR	NOD-like receptors
NO	nitric oxide
NOD	nucleotide-binding oligomerization domain
Nrf2	NFE-related factor 2
oxLDL	oxidized low-density lipoprotein
PAMP	pathogen-associated molecular pattern
PBMCs	peripheral blood mononuclear cells
PD	Parkinson’s disease
PNS	peripheral nervous system
PYRIN	amino-terminal pyrin domain
PRR	pattern recognition receptor
RA	Rheumatoid Arthritis
RE	Rasmussen’s Encephalitis
ROS	reactive oxygen species
SIRT1	Sirtuin 1
T2D	type 2 diabetes
TAC	transverse aortic constriction
TLR	Toll-like receptor
TNBS	2,4,6-trinitrobenzenesulfonic acid
TNF	tumor necrosis factor
TXNIP	thioredoxin-interacting protein
UC	ulcerative colitis
